# Ablative and Immunostimulatory Effects of Histotripsy Ablation in a Murine Osteosarcoma Model

**DOI:** 10.3390/biomedicines11102737

**Published:** 2023-10-09

**Authors:** Alayna N. Hay, Khan Mohammad Imran, Alissa Hendricks-Wenger, Jessica M. Gannon, Jacqueline Sereno, Alex Simon, Victor A. Lopez, Sheryl Coutermarsh-Ott, Eli Vlaisavljevich, Irving C. Allen, Joanne L. Tuohy

**Affiliations:** 1 Department of Small Animal Clinical Sciences, Virginia Maryland College of Veterinary Medicine, Blacksburg, VA 24061, USA; 2 Department of Biomedical Sciences and Pathobiology, Virginia Maryland College of Veterinary Medicine, Blacksburg, VA 24061, USAicallen@vt.edu (I.C.A.); 3 Translational Biology, Medicine and Health Graduate Research Program, Virginia Tech, Roanoke, VA 24016, USA; 4 Department of Biomedical Engineering and Mechanics, Virginia Tech, Blacksburg, VA 24061, USAeliv@vt.edu (E.V.); 5 Virginia Department of Agriculture and Consumer Services, Wytheville, VA 24382, USA

**Keywords:** tumor ablation, focused ultrasound, tumor immunology, DLM8 cell line

## Abstract

*Background*: Osteosarcoma (OS) is the most frequently occurring malignant bone tumor in humans, primarily affecting children and adolescents. Significant advancements in treatment options for OS have not occurred in the last several decades, and the prognosis remains grim with only a 70% rate of 5-year survival. The objective of this study was to investigate the focused ultrasound technique of histotripsy as a novel, noninvasive treatment option for OS. *Methods:* We utilized a heterotopic OS murine model to establish the feasibility of ablating OS tumors with histotripsy in a preclinical setting. We investigated the local immune response within the tumor microenvironment (TME) via immune cell phenotyping and gene expression analysis. *Findings:* We established the feasibility of ablating heterotopic OS tumors with ablation characterized microscopically by loss of cellular architecture in targeted regions of tumors. We observed greater populations of macrophages and dendritic cells within treated tumors and the upregulation of immune activating genes 72 h after histotripsy ablation. *Interpretation:* This study was the first to investigate histotripsy ablation for OS in a preclinical murine model, with results suggesting local immunomodulation within the TME. Our results support the continued investigation of histotripsy as a novel noninvasive treatment option for OS patients to improve clinical outcomes and patient prognosis.

## 1. Introduction

Osteosarcoma (OS) is the most commonly occurring primary bone tumor and primarily occurs in children and adolescents [1,2,3,4,5]. The worldwide incidence in children and adolescents is 3–4.5 cases per 1 million people and accounts for 55% of primary bone tumor diagnoses in children in the United States [3,6,7]. Over 30 years ago, chemotherapeutics were introduced into the standard of care treatment regimen for OS patients, which greatly improved patient survival. However, survival outcomes remain at a median 5-year survival rate of 70% for patients presenting without metastatic disease at the time of diagnosis, and less than 25% for patients with metastatic or recurrent disease [1,2,4,8]. The current definitive standard of care treatment for OS consists of surgical resection of the primary bone tumor via limb salvage surgery or amputation with neoadjuvant and adjuvant chemotherapy [1,9]. Advancements in limb salvage surgeries have resulted in improving quality of life outcomes for OS patients, but these procedures are still associated with complications and risks, including mobility limitations, fracture, infection, and tumor recurrence [1,9,10]. Additionally, limb salvage surgery is an invasive procedure, and often multiple surgeries are required [11,12,13]. Consequently, there is a continued need to advance OS treatment modalities in order to improve patient quality of life and survival. A noninvasive limb salvage treatment option has the potential to immensely improve patient quality of life.

In a similar manner to many cancers, the cellular interactions that occur in the tumor microenvironment (TME) in OS result in immunosuppression, contributing to OS progression and metastatic disease development, which is the major contributing factor to OS mortality [14,15,16,17]. Thus, more recent approaches for developing novel OS treatments have focused on immunomodulation in efforts to stimulate an antitumor immune response to moderate metastatic disease development [14,18,19,20,21]. The novel focused ultrasound technique, histotripsy, has been shown to ablate targeted tumor tissue and induce immunomodulation to stimulate an antitumor immune response [22,23,24,25,26,27,28,29,30,31]. Histotripsy is thus an exciting technique that can potentially treat primary and metastatic OS to improve treatment and survival outcomes.

Histotripsy is a precise, nonthermal, noninvasive, focused ultrasound technique that utilizes high-amplitude ultrasound pulses to generate acoustic cavitation to mechanically homogenize targeted tissue [31,32,33,34,35,36]. Histotripsy has previously been employed in humans to ablate prostate tissue, heart valves, and liver tumors [36,37,38,39] and in canines for experimentally induced prostate tumors [40], spontaneously occurring primary bone tumors [25,41,42,43], and soft tissue sarcomas [43,44]. Preclinical rodent models have demonstrated effective ablation of liver tumors [45], melanoma [27], renal cell carcinoma [28], and pancreatic [24] tumors and have also reported that the resulting disruption of targeted tissue causes the release of immunogenic cellular proteins, which stimulates a proinflammatory/antitumor immune response [26,27,28,29,45,46]. The destruction of tumors with histotripsy leads to the release of tumor antigens and damage-associated molecular patterns (DAMPs), such as high mobility group box-1 (HMGB-1), orchestrating an antitumor immune response [24,26,27,28]. Furthermore, an increased infiltration of cytotoxic T-lymphocytes (CTLs) into the tumor microenvironment is observed after histotripsy treatment, as is an increase in tumor antigen recognition and tumor cell cytotoxicity [23,24,26,27,28]. In longer-term preclinical studies, the abscopal immune response associated with histotripsy was reported to decrease metastases within 20 days after histotripsy treatment [27,46,47]. A clinical trial investigating histotripsy hepatic tumor ablation observed a decrease in the growth of non-histotripsy-treated tumors in study patients [47]. Histotripsy has the potential to serve as both a primary tumor ablation modality and to stimulate an antitumor immune response. However, in the context of OS, much remains to be investigated regarding the potential of employing histotripsy ablation and the associated immunomodulatory effects.

The current study was conducted to investigate the ablative and preliminary immunological outcomes associated with histotripsy ablation of OS in the syngeneic C3H/HeN mouse and DLM8 cell line models. Ablation outcomes were evaluated histologically immediately after histotripsy and 72 h after treatment. We analyzed the expression of immune activation genes *Il6*, *Ifnγ*, *Il-1β*, *Il10*, *Il13*, and *Pdl1* and characterized immune cell populations within the TME 72 h after treatment. We hypothesized that histotripsy ablation would result in disruption of the targeted OS tumors, yield acellular debris, and stimulate a proinflammatory immune response. Our results indicate successful targeted ablation of OS tumors and suggest that histotripsy modulates the TME, thus supporting the potential of histotripsy as a treatment modality for OS.

## 2. Materials and Methods

### 2.1. Murine OS Model

The syngeneic C3H/HeN mouse (RRID:MGI:2160972) DLM8 OS cell line (RRID:CVCL_6669) murine model was utilized for the current study. The DLM8 cell line was a gift from Dr. E. Kleinerman (M.D. Anderson Cancer Center). DLM8 cells were maintained in Dulbecco’s modified eagle medium with GlutaMAX (Gibco, Thermo Fisher Scientific, Waltham, MA, USA) supplemented with 10% heat-inactivated fetal bovine serum (Atlas, Fort Collins, CO, USA), 1 mM sodium pyruvate (Thermo Fisher Scientific, Waltham, MA, USA), 1% nonessential amino acid solution (Gibco, Thermo Fisher Scientific, Waltham, MA, USA), and 1% penicillin/streptomycin (Gibco, Thermo Fisher Scientific, Waltham, MA, USA). Cells were subcultured at 70–80% confluency with 1% trypsin supplemented with 0.5 mM EDTA (Gibco, Thermo Fisher Scientific, Waltham, MA, USA), and passages 14–18 were used for tumor cell injections.

Female, 6–8-week-old C3H/HeN mice (Strain Code 025, Charles River, Chapel Hill, NC, USA) weighing ~20 g were used for this study. A total of 30 tumor-bearing mice were divided into two groups for this study. The immediate group (n = 6 total) was sacrificed immediately after histotripsy treatment for the ablative outcome assessment, and the 72-h group (n = 24 total) was sacrificed 72 h after histotripsy treatment for the ablative and immunological outcome assessment. Both the immediate and 72 h groups consisted of tumor-bearing mice that were either part of untreated or histotripsy-treated cohorts. Mice were euthanized via CO_2_ inhalation. For tumor induction, mice were anesthetized with isoflurane (2.0 L/min oxygen, 3% isoflurane), and 100 µL of 2 × 10^6^/mL DLM8 tumor cells suspended in Matrigel (Corning Inc., Corning, NY, USA) was subcutaneously injected into the right flank. To monitor tumor growth, two perpendicular diameter measurements were obtained 3×/week with calipers. Histotripsy treatment was delivered when the largest tumor diameter reached an average of 7 mm amongst all study mice (immediate group n = 3 treated and 3 untreated mice and 72 h n = 12 treated mice and 12 untreated mice). Overall health, weight, and tumor growth were monitored 3×/week for the duration of the study. All experiments were conducted under Virginia Tech Institutional Animal Care and Use Committee (protocol number 21-063) approval and in accordance with the NIH Guide for the Care and Use of Laboratory Animals.

### 2.2. Histotripsy System and Ablation Parameters

**Figure 1:**
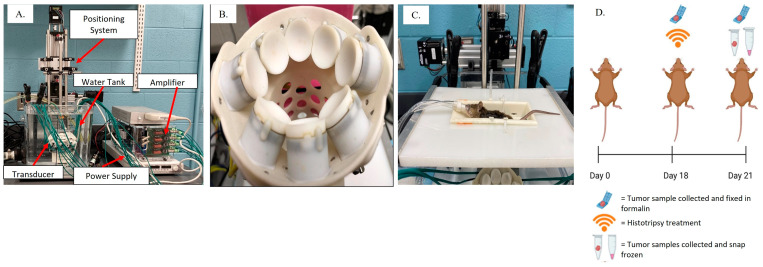
In vivo histotripsy treatment setup and timeline: (**A**) histotripsy system for small animal treatment with the major components labeled; (**B**) a 1-MHz therapy transducer with an ultrasound imaging probe coaxially aligned for real-time monitoring of treatment; (**C**) anesthetized mouse placed on the subject stage with the tumor submerged in degassed water. (**D**) Schematic of the study timeline: day 0—DLM8 tumor cells were SQ injected; day 18—histotripsy treatment was delivered to treated mice (n = 12), the immediate group was sacrificed, and tumor tissue was collected for histology (n = 3); day 21—72 h after treatment, 72 h group of mice were sacrificed, and tumor tissue was collected from flow cytometry (n = 3/group), gene expression analysis (n = 9/group), and histology (n = 9).

The histotripsy system utilized in the current study has been previously described [41,48]. Briefly, the custom system was composed of a 1MHz, 8-element small animal histotripsy transducer with a coaxially aligned linear ultrasound imaging probe with a frequency range of 10–18 MHz (L18-10L30H-4, Telemed, Vilnius, Lithuania, EU) coaxially aligned (Figure 1). The therapy transducer was driven by a custom high-voltage pulser designed to generate short single pulses of <2 cycles controlled by a field-programmable gate array (FPGA) board (Altera DE0-Nano Terasic Technology, Dover, DE, USA). The transducer was powered by a high-voltage DC power supply (GENH750W, TDK-Lambda, National City, CA, USA), positioned in a tank of degassed water heated to 37 ± 4 °C beneath a custom-designed mouse surgical stage, and attached to a computer-guided 3-D positioning system (Figure 1). The system was controlled using a custom user interface operated through MATLAB (MATLAB 2017a, MathWorks, Portola Valley, CA, USA).

Histotripsy treatment was delivered to an ellipsoidal volume within the tumor as previously described [24,48], with the exception of treatment parameters. Histotripsy was applied using short single-cycle pulses delivered at a pulse repetition frequency (PRF) of 500 Hz, with treatment points spaced 1mm in the (axial) × 0.5 mm (lateral) × 0.5 mm (vertical) with a 2 s dwell time at each treatment point for delivery of 1000 pulses/point. For this study to evaluate ablation and immune effects, partial ablations were conducted.

### 2.3. Tissue Collection and Processing

For the immediate group, tumor samples were harvested and collected from the untreated cohort (n = 3) and the histotripsy-treated cohort (n = 3) immediately following treatment and fixed in 10% formalin for histological analysis. For both the untreated cohort and the histotripsy-treated cohort in the 72 h group, tumor samples were harvested, sectioned in half, and each tumor half was either flash frozen for RNA isolation, fixed in 10% formalin for histological analysis (n = 9/cohort), or whole tumor samples were collected and placed in ice-cold 1xPBS for processing for flow cytometry (n = 3/cohort) (Figure 1D). The assessment focus for the immediate group was the histotripsy ablation zone and the innate immune response for the 72 h group.

### 2.4. Histopathology

Harvested formalin-fixed tumor samples were paraffin-embedded, sectioned into 5 µm sections, and stained with hematoxylin and eosin (H&E) for histological analysis. Tumor sections were blindly evaluated by a board-certified veterinary pathologist (S.C.O.) and assessed qualitatively and semiquantitatively. Semiquantitative assessments included a review of a single, 5 µm H&E section of tumor and the application of a scoring system to semiquantify both total tissue destruction (an indicator of mechanical destruction of cells) and tumor necrosis (an indicator of cell death). Total tissue destruction was defined by a complete loss of recognizable tissue architecture (cellular or stromal) and replacement by a fine basophilic dust. Tissue necrosis was defined as the presence of dead or dying cells with varying amounts of eosinophilic debris and clumped basophilic nuclear debris with a variably intact stroma. A score of 0–4 was assigned to each sample according to the following parameters: 0 = 0% of the tissue exhibits characteristic features of either parameter, 1 = 1–25% of the tissue exhibits characteristic features of either parameter, 2 = 26–50% of the tissue exhibits characteristic features of either parameter, 3 = 51–75% of the tissue exhibits characteristic features of either parameter, and 4 = 76–100% of the tissue exhibits characteristic features of either parameter. Additionally, samples from the 72 h group were assessed and scored for inflammatory cell infiltration.

### 2.5. Gene Expression Analysis

Total RNA was extracted from each flash frozen tumor sample with TRIzol (Invitrogen, Waltham, MA, USA) and transcribed to cDNA with a high-capacity cDNA reverse transcription kit (Thermo Fisher Scientific, Waltham, MA, USA) following the manufactures’ protocols. RT-qPCR was performed with TaqMan Fast Advanced Master Mix (Thermo Fisher Scientific, Waltham, MA, USA), and the following TaqMan probes (Thermo Fisher Scientific) were utilized: 18s (Mm04277571_s1), *Il6* (Mm00446190_m1), *Ifnγ* (Mm0116134_m1), *Il1β* (Mm00434228_m1), *Il10* (Mm01288386_m1), *Il-13* (Mm00434204_m1), and *Cd274* (Mm03048248_m1) genes. The ΔΔCt methodology was used for calculating fold change for genes of interest. The genes of interest were normalized to reference gene 18s, and the reported fold change was relative to the untreated tumor group. Technical replicates were run in triplicates.

### 2.6. Flow Cytometry Analysis 

Tumor tissues collected for flow cytometry were homogenized into a single-cell suspension via mechanical digestion. After homogenization, cell suspensions were filtered with a 70 µM cell strainer, centrifuged at 300× *g* for 10 min at 4 °C, and resuspended in 1 × PBS + 2% FBS (FACS buffer) at a concentration of 10 × 10^6^ cells/mL. For antibody staining, 1 × 10^6^ cells were plated onto a 96-well V-bottom plate, and each sample was incubated with 0.5 µg of anti-mouse CD16/32 for 1 h at 4 °C. Following this incubation period, cells were incubated with mouse-specific primary antibodies, anti-CD45 Alexa 700, anti-Ly6C efluor 450, anti-CD11b PE-Cy7, anti-CD11c PerCP-Cy5.5, anti-F4/80 PE, anti-Ly6G APC, anti-MHC-II BV510, anti-CD3 BV421, anti-CD8a BV510, anti-CD4 PerCP-Cy5.5, anti-FoxP3 APC, and anti-CD19 PE/Cyanine7 for 30 min at 4 °C in the dark. A 100 µL of FACS buffer was added to each sample, the plate was centrifuged at 300× *g* for 10 min at 4 °C, and samples were resuspended in 200 µL of FACS buffer and analyzed with a FACS Aria (BD Biosciences, Franklin Lakes, NJ, USA). Primary antibody clones and concentrations are in Table 1. All antibodies were purchased from BioLegend (San Diego, CA, USA) with the exception of Ly6C, which was purchased from ThermoFisher, and FoxP3 from R&D (Minneapolis, MN, USA). Data analysis was completed with the software Kaluza version 2.1 (Beckman Coulter, Brea, California, USA, sourced from Beckman Coulter) after gating on single cells.

### 2.7. Data and Statistical Analysis

For statistical analysis, GraphPad Prism version 8.0 was used. Comparisons between two experimental groups were performed using a Student’s two-tailed *t*-test. One-way and two-way ANOVA were utilized where multiple comparisons were conducted, and the Tukey post hoc test was used for multiple pairwise comparisons. Sample size calculations were based on previous power analyses conducted for our laboratory, and group sizes yielded a power of greater than 80%. Statistical significance was specified as *p* < 0.05. The predicted histotripsy ablation volume was calculated based on the minor and major radii of the MATLAB-generated ellipsoidal treatment volume.

## 3. Results

### 3.1. Tumor Tissue Can Be Effectively and Precisely Targeted and Ablated with Histotripsy

**Figure 2:**
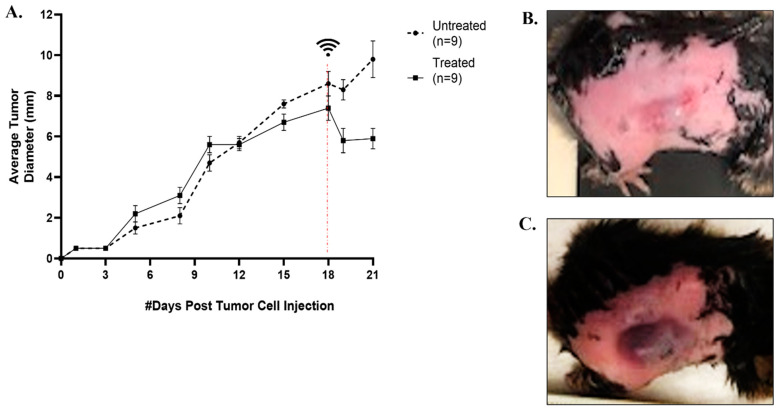
Feasibility of delivering histotripsy ablation to SQ OS tumors: (**A**) average tumor diameter in treated and untreated mice from day 1 of tumor cell injection to day 21 (72 h after histotripsy treatment); (**B**) image of SQ OS tumor on the right flank of the C3H/HeN mouse prior to histotripsy treatment; (**C**) image of tumor immediately after histotripsy treatment. Error bars are represented as SD.

**Figure 3:**
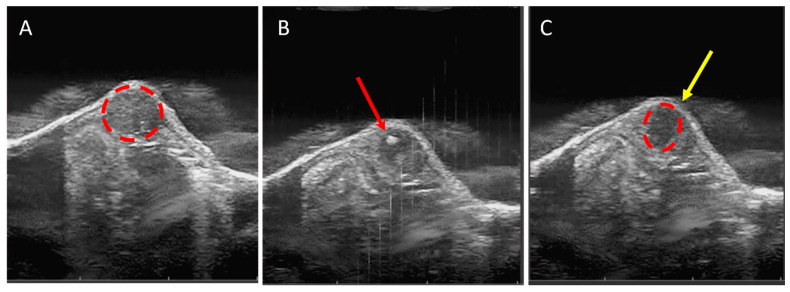
**Representative B-mode ultrasound images:** (**A**) DLM8 subcutaneous tumor before treatment (red dashed circle); (**B**) tumor during treatment with cavitation bubble cloud (red arrow); (**C**) ablated tumor tissue (red dashed circle) with hypoechoic regions (yellow arrow).

For the current study, the syngeneic C3H/HeN mouse-DLM8 cell line heterotopic SQ OS tumor model was employed to gain preliminary insight on the feasibility of ablating OS tumors with histotripsy. We hypothesized that histotripsy would need to be delivered at a dose that paralleled what we previously employed for ablating fibrotic cholangiocarcinoma liver tumors [48]. At 18 days after tumor cell injection, the tumor diameter reached an average of 7 mm (Figure 2A,B), and histotripsy treatment was delivered to mice in the immediate group (n = 3) and 72 h group (n = 12). Histotripsy treatment was delivered to an estimated ellipsoidal volume of 23 mm^3^ ± 10 mm^3^. Immediately after histotripsy treatment, edema and bruising were evident at the tumor site (Figure 2B,C). However, the edema began to resolve by 24 h after treatment, and by 72 h, manual caliper measurements indicated the tumors had reduced by 48% in size compared to before treatment. At 72 h after treatment, tumors were on average 30% smaller than untreated tumors, based on manual caliper measurements (Figure 2A). A cavitation bubble cloud was generated within the targeted tumor tissue and was consistently visualized for the duration of treatment (Figure 3A,B). Successful treatment was confirmed by hypoechoic regions within the targeted region of the tumor, which was observed with ultrasound imaging (Figure 3C).

### 3.2. Histotripsy Ablation Resulted in Effective Tumor Tissue Destruction, Necrosis, and Immune Cell Infiltration

**Figure 4:**
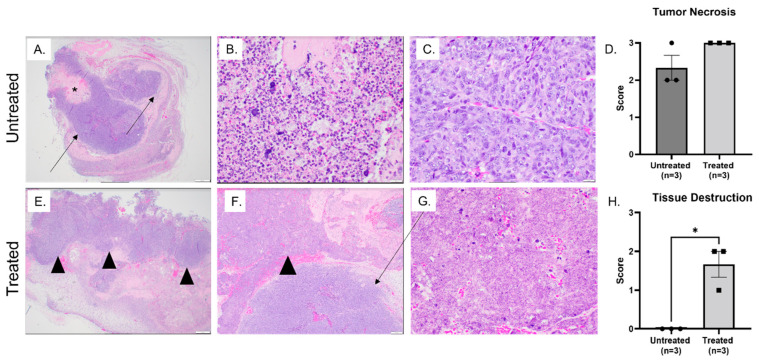
Representative H&E-stained images of tumor tissues collected immediately after histotripsy treatment: samples from untreated (**A**–**C**) and treated (**E**–**G**) tumors were evaluated for intact tumor cells and tumor necrosis; (**A**) low magnification image of untreated tumor tissue which was characterized by predominantly lytic necrosis with variable neutrophilic infiltration (asterisk) and a mixture of intact tumor cells (arrows); (**B**) high magnification image of lytic necrosis with variable neutrophilic infiltration; (**C**) high magnification image depicting intact tumor cells; (**D**) scoring of tumor necrosis in evaluated tumor samples; (**E**) low magnification image depicting large foci of total tissue destruction characterized by loss of all cellular architecture and replacement by a fine basophilic material (arrowheads); (**F**) high magnification image depicting region of tissue destruction (arrowhead); (**G**) higher magnification image depicting tissue destruction and fine basophilic material; (**H**) tissue destruction scoring. There was significantly greater tissue destruction in treated tumors compared to untreated tumors. Error bars represent SEM. * *p* = 0.04. Statistical analysis was computed with an unpaired *t*-test with Welch’s correction.

**Figure 5:**
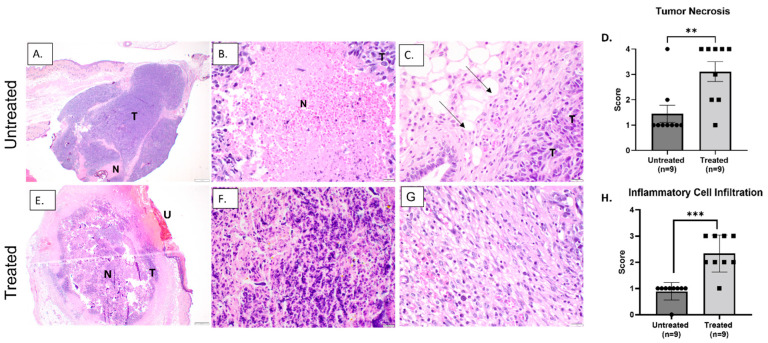
Representative H&E-stained images of tumor tissues 72 h after histotripsy treatment: untreated tumor samples (**A**–**C**) and treated tumor samples (**E**–**G**); (**A**) low magnification image depicting a mixture of intact tumor cells (T), foci of necrosis (N), and ulceration of the overlying skin in treated tumor region (U); (**B**) high magnification image depicting intact tumor cells (T) and foci of necrosis (N); (**C**) low numbers of inflammatory cells infiltrating the periphery of the tumor (arrows); (**D**) scoring of tumor necrosis in treated and untreated tumor tissue; (**E**) low magnification image depicting large foci of tumor necrosis (N) in treated tumor tissue; (**F**) high magnification image depicting foci of necrosis; (**G**) inflammatory cells expanding the periphery of treated tumors; (**H**) scoring of inflammatory cell infiltration. Error bars represent SEM. ** *p* = 0.005 and *** *p* = 0.0002. Statistical analysis was computed with an unpaired *t*-test with Welch’s correction.

Sections of harvested tumors were fixed in formalin, sectioned, and routinely stained with H&E to facilitate histological examination. Untreated tumors from the immediate and 72 h groups were characterized by a mixture of intact tumor cells and variably sized and distributed foci of tissue necrosis. In general, tissue necrosis was primarily lytic, accompanied by areas of edema and/or eosinophilic proteinaceous debris and variable neutrophil infiltration in all untreated tumors (Figure 4A–C and Figure 5A–C). Intact tumor cells were characterized by sheets of malignant spindle cells with numerous mitotic figures (Figure 4C and Figure 5B). At the periphery of the untreated tumors, a limited number of macrophages, lymphocytes, and, to a lesser extent, neutrophils were observed (Figure 5C). Untreated tumors from the 72 h group also exhibited foci of mineralization, occasional coagulative necrosis, and rare acute hemorrhage, which were not observed in the immediate group (Figure 5A–C).

Treated tumors harvested immediately after treatment exhibited greater amounts of tumor necrosis when compared to untreated tumors harvested at the same time point. Additionally, treated tumors exhibited large regions of total tissue destruction and significant acute hemorrhage. In these regions, tissue architecture was completely obliterated and replaced by a fine basophilic dust that was not observed in any untreated tumors (Figure 4E–H). The scoring of the tumor destruction regions revealed significantly (*p* = 0.04) greater tissue destruction in treated tumor samples immediately after histotripsy compared to untreated tumors (Figure 4H). Inflammatory cell infiltration was evaluated, and no major differences were observed between treated and untreated samples in the immediate group.

Treated tumors harvested 72 h after histotripsy treatment exhibited very large foci of tissue necrosis, including extensive regional ulceration of the skin overlying the tumor. The majority of tissue necrosis was lytic, with aggregates of basophilic debris marking remnants of coagulated nuclear material (Figure 5E–G). When scored and compared with untreated tumors harvested at the same time point, these differences were statistically significant (*p* = 0.005) (Figure 5D). At 72 h, scattered foci of basophilic debris identified as total tissue destruction were sporadically observed, but this was not as significant of a feature as in the immediate group. Additionally, we consistently observed significantly (*p* = 0.0002) greater numbers of mononuclear inflammatory cells, primarily macrophages and lymphocytes, at the periphery of treated tumors compared to untreated samples at 72 h after treatment (Figure 5G,H).

### 3.3. Histotripsy Ablation Induces Immune Activation within the TME

**Figure 6:**
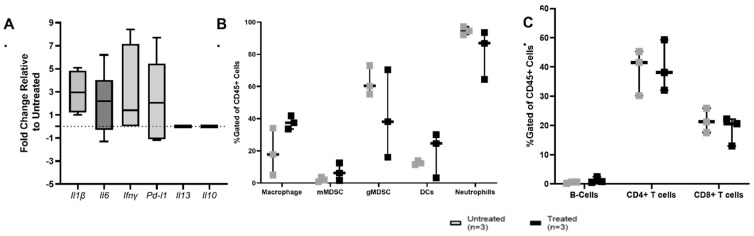
Tumor immune microenvironment evaluation: (**A**) differential gene expression analysis of immune-associated genes was calculated relative to the untreated tumor utilizing the standard ΔΔ Ct methodology; (**B**) innate and (**C**) adaptive tumor immune cell populations were evaluated 72 h after histotripsy treatment. All data are presented as box and whisker plots. Statistical analysis was calculated using a two-way ANOVA. No statistical differences were observed.

At 72 h after histotripsy treatment, we evaluated immune activation within the tumor microenvironment via differential gene expression analysis and immunophenotyping. At the time of sacrifice, tumor samples were collected and either snap frozen for future RNA extraction (n = 9/group) or fresh tumor samples were processed (n = 3/group) for immunophenotyping via flow cytometry. Relative to untreated tumors, differential gene expression analysis revealed a statistically insignificant median upregulation of proinflammatory signaling molecules *Il6*, *Ifnγ*, *Il1β*, and *Pd1l*, and the gene expression for the anti-inflammatory cytokines *Il10* and *ll13* was not within the detection limit (Figure 6A). For immunophenotyping, untreated and treated tumors (n = 3/group) were evaluated for monocytic-derived myeloid-derived suppressor cells (mMDSC) or granulocytic-derived myeloid-derived suppressor cells (gMDSC), macrophages, neutrophils, myeloid-derived dendritic cells (mDCs), T-helper-lymphocytes, cytotoxic-T-lymphocytes, and B-lymphocytes via flow cytometry. In treated mice, there was at least a 20% greater median population of macrophages, DCs, and gMDSCs in treated tumors compared to untreated tumors (Figure 6B). The changes observed in the adaptive immune cell populations were modest (Figure 6C). All observed changes were statistically insignificant.

## 4. Discussion

The application of the focused ultrasound technique histotripsy is an emerging ablation modality that is gaining increased interest for tumor ablation [36], and the results of this paper support its potential use for tumor ablation, specifically OS. Previous work performed in the focused ultrasound research field has demonstrated successful application of high-intensity focused ultrasound (HIFU) for pain palliation in patients with metastatic bone tumors [49,50,51,52]. However, the nonthermal nature of histotripsy is favored over thermal HIFU ultrasound due to decreased risks of damage to the skin overlying targeted tissue, off-targeting related to the heat sink effect, and thermal denaturation of antigens released from the targeted tumor, potentially improving the immunogenicity of the released antigens [23,25,36]. The previous reported use of HIFU for bone tumors supports the investigation of focused ultrasound for treatment of bone tumors, but the described thermal ablation limitations warrant investigation of the nonthermal focused ultrasound modality histotripsy. The treatment of bone tumors with electrochemotherapy (ECT) [53] and radiotherapy [54] has also been reported as nonthermal and nonablative techniques to reduce bone tumor burden and tumor-associated pain. However, histotripsy proposes advantages over these nonablative techniques with its potential to serve as a primary tumor removal modality and stimulate an antitumor immune response to mitigate metastatic disease and recurrent primary disease. Therefore, in the current study, we evaluated the feasibility of ablating heterotopic SQ OS tumors in a preclinical murine model with histotripsy and evaluated the innate immune response 72 h after histotripsy ablation.

We successfully achieved targeted partial ablation of SQ OS tumors with 500 Hz PRF and a treatment dose of 1000 pulses/point, as was previously used for cholangiocarcinoma ablation [48]. Histotripsy ablation was identified in real-time on ultrasound by characteristic hypoechoic regions (Figure 3C) and was also evident histologically (Figure 4E–G and Figure 5E–G). For immediate evaluation of histotripsy ablation outcomes, tumor samples were collected immediately after ablation. These tumor samples exhibited microscopic features compatible with the mechanical disintegration of cells and tissue expected from histotripsy treatment (Figure 4E–G). These features included a complete loss of tissue architecture, cellular material, and stroma, with replacement by a fine basophilic dust. There was significantly greater tissue destruction in histotripsy-treated tumors compared to untreated tumors (Figure 4H). These histological changes paralleled changes that have previously been reported to be associated with histotripsy ablation in pancreas and liver preclinical tumor models [24,48]. We observed a progressive reduction in treated tumor size for 72 h following treatment, likely predominantly due to resolving histotripsy-induced inflammation but also the reduction in tumor size (Figure 2A). At 72 h after treatment, there was evidence of tumor necrosis and infiltration of mononuclear inflammatory cells, mainly macrophages and lymphocytes, in greater amounts in treated tumor samples compared to untreated samples (Figure 5). The histological observation of inflammatory cell infiltration at 72 h supports our tumor immune microenvironment results, which demonstrated upregulation of *Il6*, *Ifnγ*, *Il1β*, and *Pd1l* genes and a greater median population of innate immune cells in treated tumors compared to untreated tumors, and further suggests immune activation after histotripsy treatment. Furthermore, the observation of greater amounts of tissue necrosis in histotripsy-ablated tumors at 72 h suggests that histotripsy treatment is associated with continued tumor cell death for up to at least 72 h following treatment. Further investigation is warranted to evaluate the long-term ablative and immunological outcomes of histotripsy treatment after 72 h. Additionally, future studies evaluating additional noninvasive tumor ablation modalities, such as thermal high-intensity focused ultrasound (HIFU), are warranted for determining the immunological and ablative outcomes that are specific to histotripsy ablation. While previous investigations have demonstrated the success of delivering histotripsy treatment to SQ tumors, including models for pancreatic tumors [24], hepatocellular carcinoma [27,45], cholangiocarcinoma [23], and melanoma [27], this is the first report of using histotripsy to ablate OS in a murine SQ model.

Evaluating the immunomodulatory response of OS to histotripsy ablation in a heterotopic SQ OS murine model is an initial step in establishing histotripsy’s ability to induce an immunomodulatory response in OS. Our results suggest that histotripsy treatment activates a local immune response within the TME at 72 h after histotripsy ablation. Our gene expression results indicated moderate upregulation of proinflammatory cytokine genes *Il1β*, *Il6*, *Ifnγ*, and immune checkpoint molecule *Pdl-1* based on median expression levels, and expression of anti-inflammatory cytokine genes *Il10* and *Il13* was not detected (Figure 6A). The cytokine IL-6 is a pleiotropic cytokine with a variety of functions, and like the immune checkpoint molecule PD-L1, is often classified as a pro-tumorigenic molecule [55,56]. However, it should be acknowledged that the upregulation of these genes does not directly correlate to increased protein production of these signaling molecules, and further investigation is warranted to unravel the effect of histotripsy ablation on cytokine production. Additionally, upregulation of the immune checkpoint molecule PD-L1 in an immunological “cold” tumor may help promote a favorable environment that supports a response to checkpoint inhibitor immunotherapy [56]. Similar to our findings, neuroblastomas treated with the boiling form of histotripsy (boiling histotripsy) demonstrated greater PDL-1 expression within the TME and decreased IL-10 and increased IFNγ systemic concentrations [57]. Previous investigations have reported synergistic effects of histotripsy ablation and checkpoint inhibitors, including CTLA-4 and CD40 [27,29].

At the cellular level, we observed a greater median percentage of macrophages within treated tumors compared to untreated tumors (Figure 6B). The proinflammatory subtype of macrophages (M1) are producers of the cytokines IL-1β and IFNγ; however, further phenotyping of cell surface markers would be required to determine if the macrophages in the treated tumor populations were of an M1 phenotype. It has previously been reported that histotripsy-treated tumor cells polarize both M0 and M2 macrophages to a pro-inflammatory M1 state in vitro [26]. The treatment of OS with proinflammatory (M1) macrophage activating agents in combination with the standard of care chemotherapy treatment plans has demonstrated improved human and canine patient outcomes [19,20,21]. Furthermore, previous investigations have reported a correlation between decreased prevalence of metastatic disease and greater infiltration of macrophages (M1 and/or M2 phenotype) within the primary TME in OS, suggesting a role of macrophage infiltration in the prevention of metastatic disease [16,58,59]. Macrophages have an influential role in determining the immunological environment within the OS TME and contribute to the recruitment and activation of key immune cells such as tumor-scavenging NK cells and antigen-presenting DCs. In our study, we also observed a greater population of mDCs in treated tumor samples compared to untreated tumors. Dendritic cells are essential for activation of T-cell-mediated immunity, a crucial component of an antitumor immune response [14,17,60,61,62]. In our study, we did not observe differences in lymphocyte populations between treated and untreated tumor samples. However, previous investigations have reported that histotripsy treatment can stimulate cytotoxic T-lymphocyte (CTL) infiltration, increase immune recognition of tumor antigens, and increases the infiltration of immune cells [23,24,27]. More specifically, 3 days after melanoma tumors were treated with histotripsy, an increase in intratumoral natural killer cells, DCs, neutrophils, B-cells, T-helper cells, and CTL populations was observed [27]. Additionally, we observed less gMDSC in treated tumors compared to untreated tumors, which is favorable for promoting an antitumor immune response given the immunosuppressive nature of these cells [63]. Collectively, these results indicate the histotripsy led to local immune activation 72 h after treatment.

The findings of the current study are valuable and informative, but the limitations should be acknowledged and used to guide future studies. The goal of the current study was to gather preliminary knowledge on the innate immune response associated with OS and histotripsy, but future studies should characterize both the systemic and local immune responses at further timepoints after histotripsy treatment. Evaluating the immune response 10 to 14 days after histotripsy will provide a further understanding of the early adaptive immune response. Additionally, long-term studies will allow for the evaluation of tissue resolution of the targeted tumor by assessing continued tumor regression or recurrence and evaluating the impact of histotripsy on metastatic disease development. All of these aspects are important to evaluate in order to further understand the ablative effects of histotripsy ablation of OS to develop histotripsy for ablating the primary OS tumor and to characterize the immunomodulatory effects of histotripsy ablation of OS to develop histotripsy for mitigating metastatic disease, which is the primary cause of death in OS patients.

In conclusion, the results of our pilot study pertaining to the ablation of SQ OS tumors demonstrate the feasibility of ablating OS tumors in this heterotopic SQ OS preclinical model, which is valuable for advancing further feasibility and safety studies in both heterotopic and orthotopic models. Moreover, our results support the immune activation potential of histotripsy by demonstrating local immune activation 72 h after histotripsy treatment. This study is the first reported preclinical rodent model study for investigating histotripsy ablation for OS. This investigation continues to support the advancement of the clinical translation of histotripsy as a noninvasive treatment option for OS, which has strong potential to improve both clinical outcomes and patient prognosis. 

## Figures and Tables

**Table 1:** Flow cytometry anti-mouse primary antibodies, clones, and working concentrations.

Antibody	Clone	Concentration
CD45	30-F11	0.25 µg/mL
CD11b	M1/70	0.156 µg/mL
Ly6C	HK1.4	0.078 µg/mL
Ly6G	1A8	0.3 µg/mL
CD11c	N418	1.25 µg/mL
MHCII	M5/114.15.2	0.2 µg/mL
F4/80	BM8	2.5 µg/mL
CD3	17A2	1.25 µg/mL
CD4	GK1.5	0.156 µg/mL
CD8a	53-6.7	0.625 µg/mL
CD19	6D5	0.25 µg/mL
FoxP3	1054C	0.25 µg/mL

## Data Availability

The data that support the findings of this study are available from the corresponding author, [J.L.T.], upon reasonable request. The data are not publicly available due to privacy.
